# Notes and News

**Published:** 1892-12-10

**Authors:** 


### Notes and News.

COLLECTED AND COMMUNICATED.

The Lord Mayor has kindly consented to preside at the festival dinner, to be held at the Holborn Restaurant on May 9th next, in aid of the funds of the North-Eastern Hospital for Children, Hackney Road, N.E.

The site of the proposed new cholera hospital has been discussed in the report of the Medical Officer of Health for the City of London. There seems to be difficulties in the case of all three localities at present suggested. Should these prove insurmountable, a floating hospital may possibly be resorted to, moored between London Bridge and Waterloo Bridge.

Mr. George Trimmer, a benevolent Surrey gentleman, has recently died, and has in his will bequeathed large sums for charitable purposes. The Surrey County Hospital benefits to the extent of £500, and he has desired that a cottage hospital shall be established at Farnham, for which purpose he bequeathes £15,000. It is proposed to name the new institution the Trimmer Cottage Hospital. It is expected that Mr. Trimmer's sons will present the site.

A representative meeting of Governors of the well-known Royal Sea Bathing Infirmary for Scrofula was held on Monday, the 14th ult., at the offices, 30, Charing Cross, Mr. C. A. Swinburne, Vice-President, in the chair, when some necessary alterations were made in the rules. It has been found that in the present day the clergy, while willing to support all charitable effort, could not obtain such large sums as in former years. The old rule whereby thirty guineas or more had to be collected before any privileges would be conferred through advocating the claims of this institution from the pulpit was therefore altered, and a more reasonable sum, viz., five guineas, substituted. We think this an eminently wise course to adopt. Many a country congregation would be glad to assist, but at the same time would like to know, that should any of its poorer members require treatment at the R.S.B.I. there would be, during the succeeding five years, a means of obtaining the benefits of the infirmary without asking for outside aid. Amongst others who supported the Chairman we noted the Rev. Prebendary Whittington (Vice-President), Henry Maudsley, Esq., W. H. Dickinson, Esq., M.D., John Croft, Esq., P. S. Easton, Esq., H. Spencer Smith, Esq., all well-known in the hospital and charitable world, and all taking a great interest in the institution. We regret to learn that owing to the present needy condition of the infirmary the number of beds is to be reduced to 80.

An important meeting is to be held in the Kensington Town Hall on Monday, December 12th, at eight p.m., to give information and create interest in the "Church House and its work." The speakers will be the Bishop of Rochester, Earl Nelson, Prebendary H. Wace, D.D., and the Hon. E. P. Thesiger, C.B. We many of us are familiar with the title "Church House" without precisely understanding the "why and wherefore" of such an institution. Many of us, therefore, will welcome an occasion on which our interest will undoubtedly be awakened and our desire for information satisfied. The Church House commenced its scheme of activity almost immediately after the Jubilee, suitable buildings to commence with being found in Dean's Yard, Westminster. The Institution is practically a bureau of information on Church matters. It already possesses an excellent reference library, which should afford those who possess valuable theological books, for which they have no use, an opportunity of benefiting many. A great meeting hall, with offices necessary for the purpose, is already in course of erection, and will no doubt be the scene of many interesting gatherings in the future. Further, the scheme of the Committee embraces nursing and convalescent homes for the clergy and their families. A nursing home in Devonshire Street has been working some time, the success of which demands an extension of the premises. The cost of residence is made as low as possible, to meet the wants of individual cases, commencing with a charge of ten shillings. Free admission, even, is granted in some cases. The convalescent home will be started in the country as soon as funds permit. These two last-named projects of the Church House, attended with special difficulties, as can be readily understood, will, if these difficulties be surmounted, redound greatly to the credit of the management, and prove not the least valuable undertakings of the whole scheme.

That "extremes meet" is a well-known adage. We happened to have in our hands to-day two widely different specimens of literature, one an account of a savage people, the other a well-known modern ladies' journal, and we found to our astonishment that between the ways of savages and ladies there is undoubtedly much in common. Turning to the record of barbarism we read: "These savages devote much time to the elaborate painting of their faces." *The Lady*, in a long and "seasonable" article, as a contemporary designates it, remarks: "Belladonna is most dangerous put into the eye, however largely diluted; and although it might be used, just once for a *bal poudre*, yet the effect is so marked that the practice would probably be continued, and the sight irretrievably affected. A little eau de Cologne put in the nostrils brightens the eye without danger, and is less painful than when placed in the eye, where it often imparts a bloodshot appearance to the eye balls." Further, we read (in the journal of civilisation): "Dry irritable complexions should be carefully toned down with a very hard make of *creme l'Imperatrice*, selecting a deep tint, which will go paler by artificial light, and dusting with tinted powder. This conceals any veins, eruptions, or scars." "The lips should be tinted inside as well as out with pure liquid rouge," and much more *ad nauseam*. The ladies will learn, if for the first time through our record of the savage, that they are not first in the field. But might we remind them that health is the best preservative of beauty, and if they would follow a nineteenth century prescription, and instead of dipping into the savage grease pot, fill their lungs with pure fresh air and follow the laws of hygiene, we should not see, as we too frequently do, beings in our midst who bring to our mind the clown and the savage rather than fair women, beautiful according to nature.

